# Chloridobis{*N*-[(dimethyl­amino)dimethyl­silyl]-2,6-dimethyl­anilido-κ^2^ *N*,*N*′}iron(III)

**DOI:** 10.1107/S1600536808018114

**Published:** 2008-06-19

**Authors:** Juan Chen

**Affiliations:** a Department of Chemistry, Taiyuan Normal University, Taiyuan 030031, People’s Republic of China

## Abstract

The title iron(III) compound, [Fe(C_12_H_21_N_2_Si)_2_Cl], is monomeric. The Fe atom is *N*,*N*′-chelated by the *N*-silylated anilide ligand. The two ligands around the Fe atom are arranged *trans* to each other. The Fe—N_amino_ bond is longer than the Fe—N_anilide_ bond by about 0.37 Å. The mol­ecule displays a pseudo-twofold rotation. The five–coordinate Fe atom demonstrates a highly distorted trigonal–bipyramidal geometry.

## Related literature

For related chelate iron(III) compounds and their applications, involving, for example, porphyrin, bypyridine, amidinate as well as guanidinate, see: Rath *et al.* (2004 ▶); Schunemann *et al.* (1999 ▶); Collomb *et al.* (1999 ▶); O’Keefe *et al.* (2002 ▶); Foley *et al.* (2000 ▶). For related zinc compounds with analogous analido ligands, see: Schumann *et al.* (2000 ▶).
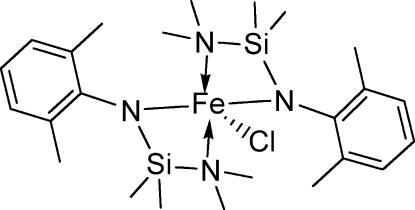


## Experimental

### 

#### Crystal data

[Fe(C_12_H_21_N_2_Si)_2_Cl]
*M* *_r_* = 534.10
Monoclinic, 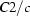

*a* = 34.213 (5) Å
*b* = 9.3555 (14) Å
*c* = 20.769 (4) Å
β = 122.924 (5)°
*V* = 5580.1 (16) Å^3^
*Z* = 8
Mo *K*α radiation
μ = 0.74 mm^−1^
*T* = 293 (2) K
0.30 × 0.25 × 0.20 mm

#### Data collection

Bruker SMART area-detector diffractometer
Absorption correction: multi-scan (*SADABS*; Sheldrick, 1996 ▶) *T*_min_ = 0.808, *T*_max_ = 0.866
11193 measured reflections
4880 independent reflections
4359 reflections with *I* > 2σ(*I*)
*R*_int_ = 0.031

#### Refinement

*R*[*F* ^2^ > 2σ(*F* ^2^)] = 0.038
*wR*(*F* ^2^) = 0.102
*S* = 1.06
4880 reflections
301 parameters
H-atom parameters constrained
Δρ_max_ = 0.48 e Å^−3^
Δρ_min_ = −0.25 e Å^−3^

### 

Data collection: *SMART* (Bruker, 2000 ▶); cell refinement: *SAINT* (Bruker, 2000 ▶); data reduction: *SAINT*; program(s) used to solve structure: *SHELXS97* (Sheldrick, 2008 ▶); program(s) used to refine structure: *SHELXL97* (Sheldrick, 2008 ▶); molecular graphics: *SHELXTL/PC* (Sheldrick, 2008 ▶); software used to prepare material for publication: *SHELXL97*.

## Supplementary Material

Crystal structure: contains datablocks I, global. DOI: 10.1107/S1600536808018114/rk2098sup1.cif

Structure factors: contains datablocks I. DOI: 10.1107/S1600536808018114/rk2098Isup2.hkl

Additional supplementary materials:  crystallographic information; 3D view; checkCIF report

## Figures and Tables

**Fig. 1.**
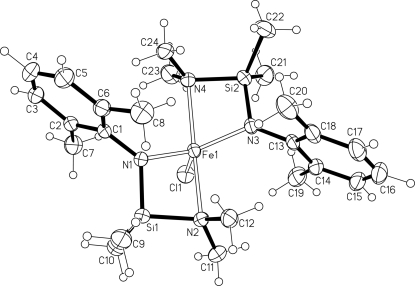
The molecular structure, showing the atom–numbering scheme. Displacement ellipsoids are drawn at the 30% probability level. H atoms are presented as a small spheres of arbitrary radius.

## supplementary crystallographic information

### Comment

The study of iron(III) amides with the monodentate ligands was relatively less because it was generally believed that a "mismatch" between the "hard" amido ligand and the "soft" late transition metal center rendered the corresponding *M*—N bond relatively unstable. Iron(III) ion could be stablized by the chelate ligands, such as porphyrin (Rath *et al.*, 2004; Schunemann *et al.*, 1999), bypyridine (Collomb *et al.*, 1999) and amidinate (O'Keefe *et al.*, 2002) as well as guanidinate (Foley *et al.*, 2000).

The title compound is supported by the *N*–silylated anilido ligand with a pendant amino group. It is the first example of iron(III) ion coordinated by an N—Si—N chelating moiety. It is monomeric and contains two *N*–silylated anilido ligands, which are arranged in *trans*– to each other and obey the *pseudo*–*C_2_* symmetrical operation. Such arrangement makes Fe atom right in the triangular planes of N1···N3···Cl1 and N2···N4···Cl1. The five–coordinate iron(III) center demonstrates a highly distorted trigonal bipyramid geometry (N2 and N4 - apical atoms), which is closely similar to the amidinate and guanidinate iron(III) compounds, but significantly different from the tetragonal pyramid geometry in the porphyrin derivatives. The Fe center is chelated, with an average N—Fe—N bite angle of 74.29 (7)°. The corresponding N—Si—N of the ligand is constrained to be about 95.49 (9)°. The two values are quite different from those in the related amidinate and guanidinate Fe(III) compounds bearing the same geometry, N—Fe—N being about 66° and N—C—N being larger than 111°. The mean Fe—N_anilido_ bond is 1.9409 (18)Å, whereas the mean Fe—N_amino_ bond is 2.3126 (19)Å in the title compound. In the reported amidinate and guanidinate iron(III) compounds, the Fe—N bonds are ranging in the scope of 2.0~2.1Å. It suggests that the N—Si—N group is more flexible in coordination chemistry, than the N—C—N chelating unit.

### Experimental

FeCl_3_ (0.21 g, 1.29 mmol) was added into the solution of [LiN(Si*Me*_2_N*Me*_2_)(2,6–*Me*_2_C_6_H_3_)]_2_ (0.59 g, 1.29 mmol) in *Et*_2_O (25 ml) at 273 K. The reaction mixture was warmed to room temperature and kept stirring for 12 h. It was dried in vacuum to remove all volatiles and the residue was extracted with CH_2_Cl_2_ (25 ml). Concentration of the filtrate under reduced pressure gave the black solid. Recrystallization of the solid in toluene yielded the title compound as black crystals (yield 0.41 g, 60%).

### Refinement

The methyl H atoms were then constrained to an ideal geometry, with C—H distances of 0.96 Å and *U*_iso_(H) = 1.5*U*_eq_(C), but each methyl group was allowed to rotate freely about its C–C bond. The other H atoms were placed in geometrically idealized positions and constrained to ride on their parent atoms, with C—H distances in the range 0.93Å and *U*_iso_(H) = 1.2*U*_eq_(C).

### Crystal data

**Table:**

[Fe(C_12_H_21_N_2_Si)_2_Cl]	*F*_000_ = 2280
*M**_r_* = 534.10	*D*_x_ = 1.271 Mg m^−^^3^
Monoclinic, *C*2/*c*	Mo *K*α radiation λ = 0.71073 Å
Hall symbol: -C 2yc	Cell parameters from 7222 reflections
*a* = 34.213 (5) Å	θ = 2.3–27.6º
*b* = 9.3555 (14) Å	µ = 0.74 mm^−^^1^
*c* = 20.769 (4) Å	*T* = 293 (2) K
β = 122.924 (5)º	Prism, black
*V* = 5580.1 (16) Å^3^	0.30 × 0.25 × 0.20 mm
*Z* = 8	

### Data collection

**Table:**

Bruker SMART area-detector diffractometer	4880 independent reflections
Radiation source: Fine–focus sealed tube	4359 reflections with *I* > 2σ(*I*)
Monochromator: Graphite	*R*_int_ = 0.031
*T* = 293(2) K	θ_max_ = 25.0º
φ and ω scans	θ_min_ = 2.0º
Absorption correction: multi-scan(SADABS; Sheldrick, 1996)	*h* = −40→33
*T*_min_ = 0.808, *T*_max_ = 0.866	*k* = −11→11
11193 measured reflections	*l* = −24→24

### Refinement

**Table:**

Refinement on *F*^2^	Secondary atom site location: Difmap
Least-squares matrix: Full	Hydrogen site location: Geom
*R*[*F*^2^ > 2σ(*F*^2^)] = 0.038	H-atom parameters constrained
*wR*(*F*^2^) = 0.102	*w* = 1/[σ^2^(*F*_o_^2^) + (0.0505*P*)^2^ + 4.0594*P*] where *P* = (*F*_o_^2^ + 2*F*_c_^2^)/3
*S* = 1.06	(Δ/σ)_max_ < 0.001
4880 reflections	Δρ_max_ = 0.48 e Å^−^^3^
301 parameters	Δρ_min_ = −0.25 e Å^−^^3^
Primary atom site location: Direct	Extinction correction: none

### Special details

**Table:**

Geometry. All s.u.'s (except the s.u. in the dihedral angle between two l.s. planes) are estimated using the full covariance matrix. The cell s.u.'s are taken into account individually in the estimation of s.u.'s in distances, angles and torsion angles; correlations between s.u.'s in cell parameters are only used when they are defined by crystal symmetry. An approximate (isotropic) treatment of cell s.u.'s is used for estimating s.u.'s involving l.s. planes.
Refinement. Refinement of *F*^2^ against ALL reflections. The weighted *R*–factor *wR* and goodness of fit *S* are based on *F*^2^, conventional *R*–factors *R* are based on *F*, with *F* set to zero for negative *F*^2^. The threshold expression of *F*^2^ > σ(*F*^2^) is used only for calculating *R*–factors(gt) *etc*. and is not relevant to the choice of reflections for refinement. *R*–factors based on *F*^2^ are statistically about twice as large as those based on *F*, and *R*–factors based on ALL data will be even larger.

### Fractional atomic coordinates and isotropic or equivalent isotropic displacement parameters (Å^2^)

**Table:**

	*x*	*y*	*z*	*U*_iso_*/*U*_eq_	
Fe1	0.152240 (10)	0.34361 (3)	0.065107 (16)	0.03404 (12)	
Cl1	0.22982 (2)	0.30852 (7)	0.13642 (4)	0.05195 (17)	
Si1	0.14458 (2)	0.46111 (7)	0.18176 (3)	0.04116 (17)	
Si2	0.12728 (2)	0.23365 (7)	−0.07896 (3)	0.04149 (17)	
N1	0.12489 (6)	0.31269 (19)	0.12496 (10)	0.0340 (4)	
N2	0.15405 (6)	0.5619 (2)	0.11810 (10)	0.0406 (4)	
N3	0.12899 (6)	0.3964 (2)	−0.04016 (10)	0.0378 (4)	
N4	0.13464 (7)	0.1293 (2)	−0.00134 (11)	0.0429 (5)	
C1	0.10023 (8)	0.1984 (2)	0.13210 (11)	0.0353 (5)	
C2	0.12431 (9)	0.0830 (3)	0.18117 (12)	0.0436 (5)	
C3	0.09884 (11)	−0.0285 (3)	0.18479 (15)	0.0602 (7)	
H3	0.1146	−0.1061	0.2165	0.072*	
C4	0.05127 (12)	−0.0272 (3)	0.14302 (17)	0.0735 (9)	
H4	0.0349	−0.1021	0.1471	0.088*	
C5	0.02808 (10)	0.0852 (4)	0.09519 (17)	0.0655 (8)	
H5	−0.0043	0.0856	0.0664	0.079*	
C6	0.05161 (8)	0.1980 (3)	0.08872 (14)	0.0454 (6)	
C7	0.17639 (9)	0.0764 (3)	0.22785 (15)	0.0592 (7)	
H7A	0.1884	0.1382	0.2716	0.089*	
H7B	0.1880	0.1067	0.1971	0.089*	
H7C	0.1862	−0.0200	0.2449	0.089*	
C8	0.02459 (9)	0.3161 (3)	0.03210 (17)	0.0633 (8)	
H8A	−0.0076	0.2892	0.0003	0.095*	
H8B	0.0369	0.3318	0.0007	0.095*	
H8C	0.0273	0.4022	0.0594	0.095*	
C9	0.10149 (11)	0.5424 (3)	0.19894 (18)	0.0663 (8)	
H9A	0.0733	0.5628	0.1507	0.099*	
H9B	0.1140	0.6295	0.2275	0.099*	
H9C	0.0950	0.4770	0.2276	0.099*	
C10	0.20056 (10)	0.4460 (3)	0.27514 (14)	0.0636 (8)	
H10A	0.1963	0.3900	0.3096	0.095*	
H10B	0.2114	0.5396	0.2963	0.095*	
H10C	0.2231	0.4004	0.2680	0.095*	
C11	0.19600 (10)	0.6507 (3)	0.15052 (17)	0.0588 (7)	
H11A	0.1986	0.6870	0.1098	0.088*	
H11B	0.2229	0.5940	0.1846	0.088*	
H11C	0.1939	0.7290	0.1783	0.088*	
C12	0.11329 (10)	0.6484 (3)	0.06259 (14)	0.0519 (6)	
H12A	0.1105	0.7291	0.0884	0.078*	
H12B	0.0856	0.5911	0.0406	0.078*	
H12C	0.1173	0.6814	0.0227	0.078*	
C13	0.12223 (9)	0.5265 (3)	−0.07968 (12)	0.0423 (5)	
C14	0.16070 (10)	0.6008 (3)	−0.07176 (14)	0.0513 (6)	
C15	0.15268 (13)	0.7255 (3)	−0.11318 (18)	0.0683 (8)	
H15	0.1779	0.7744	−0.1081	0.082*	
C16	0.10921 (15)	0.7785 (3)	−0.16087 (19)	0.0781 (10)	
H16	0.1047	0.8606	−0.1894	0.094*	
C17	0.07203 (13)	0.7101 (3)	−0.16673 (16)	0.0698 (9)	
H17	0.0424	0.7488	−0.1981	0.084*	
C18	0.07738 (9)	0.5847 (3)	−0.12716 (13)	0.0518 (6)	
C19	0.20951 (10)	0.5485 (4)	−0.02041 (18)	0.0703 (8)	
H19A	0.2145	0.4681	−0.0438	0.106*	
H19B	0.2146	0.5202	0.0280	0.106*	
H19C	0.2308	0.6236	−0.0124	0.106*	
C20	0.03569 (10)	0.5156 (4)	−0.13425 (16)	0.0689 (8)	
H20A	0.0120	0.5862	−0.1486	0.103*	
H20B	0.0444	0.4740	−0.0859	0.103*	
H20C	0.0240	0.4424	−0.1728	0.103*	
C21	0.17490 (11)	0.1969 (4)	−0.09351 (19)	0.0697 (8)	
H21A	0.1721	0.2593	−0.1325	0.105*	
H21B	0.1731	0.0993	−0.1092	0.105*	
H21C	0.2043	0.2130	−0.0464	0.105*	
C22	0.07192 (11)	0.1936 (4)	−0.17116 (15)	0.0676 (8)	
H22A	0.0464	0.1994	−0.1642	0.101*	
H22B	0.0734	0.0991	−0.1878	0.101*	
H22C	0.0674	0.2618	−0.2092	0.101*	
C23	0.17204 (12)	0.0207 (3)	0.03273 (17)	0.0708 (9)	
H23A	0.1764	−0.0137	0.0798	0.106*	
H23B	0.2005	0.0628	0.0430	0.106*	
H23C	0.1635	−0.0575	−0.0024	0.106*	
C24	0.09130 (11)	0.0623 (3)	−0.01730 (16)	0.0653 (8)	
H24A	0.0824	−0.0128	−0.0542	0.098*	
H24B	0.0670	0.1328	−0.0373	0.098*	
H24C	0.0962	0.0233	0.0293	0.098*	

### Atomic displacement parameters (Å^2^)

**Table:**

	*U*^11^	*U*^22^	*U*^33^	*U*^12^	*U*^13^	*U*^23^
Fe1	0.03341 (19)	0.0380 (2)	0.02983 (18)	0.00023 (12)	0.01659 (14)	0.00240 (12)
Cl1	0.0352 (3)	0.0610 (4)	0.0520 (4)	0.0044 (3)	0.0187 (3)	0.0056 (3)
Si1	0.0515 (4)	0.0366 (4)	0.0383 (3)	−0.0044 (3)	0.0262 (3)	−0.0041 (3)
Si2	0.0463 (4)	0.0460 (4)	0.0349 (3)	−0.0003 (3)	0.0238 (3)	−0.0015 (3)
N1	0.0364 (10)	0.0324 (10)	0.0327 (9)	−0.0003 (7)	0.0184 (8)	0.0014 (7)
N2	0.0453 (11)	0.0340 (10)	0.0378 (10)	−0.0027 (8)	0.0195 (9)	−0.0011 (8)
N3	0.0398 (10)	0.0421 (11)	0.0309 (9)	0.0008 (8)	0.0188 (8)	0.0046 (8)
N4	0.0540 (12)	0.0366 (11)	0.0417 (10)	−0.0031 (9)	0.0283 (10)	−0.0028 (8)
C1	0.0443 (13)	0.0349 (12)	0.0311 (10)	−0.0026 (9)	0.0235 (10)	−0.0021 (9)
C2	0.0594 (15)	0.0361 (13)	0.0357 (11)	−0.0013 (11)	0.0262 (11)	0.0001 (10)
C3	0.090 (2)	0.0411 (15)	0.0479 (14)	−0.0125 (14)	0.0362 (15)	0.0017 (12)
C4	0.094 (2)	0.066 (2)	0.0643 (18)	−0.0380 (18)	0.0450 (18)	−0.0039 (16)
C5	0.0549 (16)	0.082 (2)	0.0620 (17)	−0.0256 (15)	0.0330 (14)	−0.0072 (16)
C6	0.0433 (13)	0.0517 (15)	0.0446 (13)	−0.0067 (11)	0.0261 (11)	−0.0054 (11)
C7	0.0598 (17)	0.0522 (17)	0.0521 (15)	0.0127 (13)	0.0217 (13)	0.0133 (12)
C8	0.0403 (15)	0.072 (2)	0.0651 (18)	0.0058 (13)	0.0207 (13)	0.0056 (15)
C9	0.093 (2)	0.0542 (18)	0.0779 (19)	0.0006 (15)	0.0636 (19)	−0.0101 (15)
C10	0.0754 (19)	0.0621 (18)	0.0380 (13)	−0.0143 (15)	0.0210 (13)	−0.0044 (12)
C11	0.0670 (18)	0.0471 (16)	0.0627 (17)	−0.0190 (13)	0.0355 (15)	−0.0070 (13)
C12	0.0638 (17)	0.0430 (15)	0.0462 (14)	0.0108 (12)	0.0281 (13)	0.0065 (11)
C13	0.0580 (15)	0.0417 (14)	0.0306 (11)	0.0025 (11)	0.0263 (11)	0.0014 (9)
C14	0.0726 (18)	0.0434 (14)	0.0493 (14)	−0.0058 (13)	0.0406 (14)	−0.0016 (11)
C15	0.114 (3)	0.0445 (17)	0.0706 (19)	−0.0082 (17)	0.066 (2)	0.0001 (14)
C16	0.131 (3)	0.0469 (18)	0.067 (2)	0.010 (2)	0.060 (2)	0.0141 (15)
C17	0.093 (2)	0.0611 (19)	0.0468 (15)	0.0274 (17)	0.0328 (16)	0.0147 (14)
C18	0.0639 (16)	0.0548 (16)	0.0344 (12)	0.0122 (13)	0.0251 (12)	0.0048 (11)
C19	0.0643 (18)	0.073 (2)	0.079 (2)	−0.0185 (16)	0.0427 (17)	0.0017 (16)
C20	0.0528 (16)	0.090 (2)	0.0506 (15)	0.0207 (16)	0.0198 (13)	0.0096 (15)
C21	0.086 (2)	0.069 (2)	0.084 (2)	0.0023 (16)	0.0652 (19)	−0.0030 (16)
C22	0.070 (2)	0.072 (2)	0.0424 (14)	−0.0015 (15)	0.0191 (14)	−0.0119 (14)
C23	0.100 (2)	0.0492 (17)	0.0574 (16)	0.0246 (16)	0.0392 (17)	0.0058 (13)
C24	0.088 (2)	0.0653 (19)	0.0614 (16)	−0.0338 (16)	0.0529 (16)	−0.0209 (14)

### Geometric parameters (Å, °)

**Table:**

Fe1—N3	1.9406 (17)	C10—H10A	0.9600
Fe1—N1	1.9412 (18)	C10—H10B	0.9600
Fe1—Cl1	2.2523 (7)	C10—H10C	0.9600
Fe1—N2	2.3050 (19)	C11—H11A	0.9600
Fe1—N4	2.3203 (19)	C11—H11B	0.9600
Si1—N1	1.7058 (18)	C11—H11C	0.9600
Si1—N2	1.791 (2)	C12—H12A	0.9600
Si1—C10	1.847 (3)	C12—H12B	0.9600
Si1—C9	1.858 (3)	C12—H12C	0.9600
Si2—N3	1.709 (2)	C13—C18	1.407 (3)
Si2—N4	1.782 (2)	C13—C14	1.417 (4)
Si2—C21	1.844 (3)	C14—C15	1.386 (4)
Si2—C22	1.858 (3)	C14—C19	1.493 (4)
N1—C1	1.419 (3)	C15—C16	1.355 (5)
N2—C11	1.467 (3)	C15—H15	0.9300
N2—C12	1.477 (3)	C16—C17	1.369 (5)
N3—C13	1.415 (3)	C16—H16	0.9300
N4—C24	1.472 (3)	C17—C18	1.386 (4)
N4—C23	1.478 (3)	C17—H17	0.9300
C1—C6	1.396 (3)	C18—C20	1.499 (4)
C1—C2	1.404 (3)	C19—H19A	0.9600
C2—C3	1.387 (4)	C19—H19B	0.9600
C2—C7	1.497 (4)	C19—H19C	0.9600
C3—C4	1.366 (4)	C20—H20A	0.9600
C3—H3	0.9300	C20—H20B	0.9600
C4—C5	1.366 (4)	C20—H20C	0.9600
C4—H4	0.9300	C21—H21A	0.9600
C5—C6	1.377 (4)	C21—H21B	0.9600
C5—H5	0.9300	C21—H21C	0.9600
C6—C8	1.508 (4)	C22—H22A	0.9600
C7—H7A	0.9600	C22—H22B	0.9600
C7—H7B	0.9600	C22—H22C	0.9600
C7—H7C	0.9600	C23—H23A	0.9600
C8—H8A	0.9600	C23—H23B	0.9600
C8—H8B	0.9600	C23—H23C	0.9600
C8—H8C	0.9600	C24—H24A	0.9600
C9—H9A	0.9600	C24—H24B	0.9600
C9—H9B	0.9600	C24—H24C	0.9600
C9—H9C	0.9600		
			
N3—Fe1—N1	135.57 (8)	H9A—C9—H9C	109.5
N3—Fe1—Cl1	113.14 (6)	H9B—C9—H9C	109.5
N1—Fe1—Cl1	111.29 (6)	Si1—C10—H10A	109.5
N3—Fe1—N2	101.58 (7)	Si1—C10—H10B	109.5
N1—Fe1—N2	73.95 (7)	H10A—C10—H10B	109.5
Cl1—Fe1—N2	95.68 (5)	Si1—C10—H10C	109.5
N3—Fe1—N4	74.63 (7)	H10A—C10—H10C	109.5
N1—Fe1—N4	101.10 (7)	H10B—C10—H10C	109.5
Cl1—Fe1—N4	95.59 (5)	N2—C11—H11A	109.5
N2—Fe1—N4	168.71 (7)	N2—C11—H11B	109.5
N1—Si1—N2	94.59 (9)	H11A—C11—H11B	109.5
N1—Si1—C10	117.27 (12)	N2—C11—H11C	109.5
N2—Si1—C10	108.36 (11)	H11A—C11—H11C	109.5
N1—Si1—C9	114.20 (12)	H11B—C11—H11C	109.5
N2—Si1—C9	114.01 (12)	N2—C12—H12A	109.5
C10—Si1—C9	107.93 (15)	N2—C12—H12B	109.5
N3—Si2—N4	96.30 (9)	H12A—C12—H12B	109.5
N3—Si2—C21	115.89 (13)	N2—C12—H12C	109.5
N4—Si2—C21	110.08 (13)	H12A—C12—H12C	109.5
N3—Si2—C22	114.84 (12)	H12B—C12—H12C	109.5
N4—Si2—C22	112.69 (13)	C18—C13—N3	120.8 (2)
C21—Si2—C22	106.86 (15)	C18—C13—C14	118.8 (2)
C1—N1—Si1	125.09 (14)	N3—C13—C14	120.4 (2)
C1—N1—Fe1	134.44 (14)	C15—C14—C13	118.9 (3)
Si1—N1—Fe1	100.08 (9)	C15—C14—C19	119.1 (3)
C11—N2—C12	108.7 (2)	C13—C14—C19	122.0 (2)
C11—N2—Si1	118.92 (16)	C16—C15—C14	122.0 (3)
C12—N2—Si1	113.07 (16)	C16—C15—H15	119.0
C11—N2—Fe1	119.09 (16)	C14—C15—H15	119.0
C12—N2—Fe1	110.11 (14)	C15—C16—C17	119.5 (3)
Si1—N2—Fe1	85.25 (8)	C15—C16—H16	120.3
C13—N3—Si2	122.68 (14)	C17—C16—H16	120.3
C13—N3—Fe1	135.24 (15)	C16—C17—C18	121.7 (3)
Si2—N3—Fe1	101.10 (9)	C16—C17—H17	119.1
C24—N4—C23	108.6 (2)	C18—C17—H17	119.1
C24—N4—Si2	113.57 (17)	C17—C18—C13	119.1 (3)
C23—N4—Si2	117.99 (17)	C17—C18—C20	119.7 (3)
C24—N4—Fe1	113.94 (16)	C13—C18—C20	121.3 (2)
C23—N4—Fe1	115.57 (16)	C14—C19—H19A	109.5
Si2—N4—Fe1	85.87 (8)	C14—C19—H19B	109.5
C6—C1—C2	119.3 (2)	H19A—C19—H19B	109.5
C6—C1—N1	120.2 (2)	C14—C19—H19C	109.5
C2—C1—N1	120.5 (2)	H19A—C19—H19C	109.5
C3—C2—C1	118.7 (2)	H19B—C19—H19C	109.5
C3—C2—C7	119.7 (2)	C18—C20—H20A	109.5
C1—C2—C7	121.6 (2)	C18—C20—H20B	109.5
C4—C3—C2	121.7 (3)	H20A—C20—H20B	109.5
C4—C3—H3	119.1	C18—C20—H20C	109.5
C2—C3—H3	119.1	H20A—C20—H20C	109.5
C5—C4—C3	119.3 (3)	H20B—C20—H20C	109.5
C5—C4—H4	120.4	Si2—C21—H21A	109.5
C3—C4—H4	120.4	Si2—C21—H21B	109.5
C4—C5—C6	121.4 (3)	H21A—C21—H21B	109.5
C4—C5—H5	119.3	Si2—C21—H21C	109.5
C6—C5—H5	119.3	H21A—C21—H21C	109.5
C5—C6—C1	119.6 (2)	H21B—C21—H21C	109.5
C5—C6—C8	119.6 (2)	Si2—C22—H22A	109.5
C1—C6—C8	120.8 (2)	Si2—C22—H22B	109.5
C2—C7—H7A	109.5	H22A—C22—H22B	109.5
C2—C7—H7B	109.5	Si2—C22—H22C	109.5
H7A—C7—H7B	109.5	H22A—C22—H22C	109.5
C2—C7—H7C	109.5	H22B—C22—H22C	109.5
H7A—C7—H7C	109.5	N4—C23—H23A	109.5
H7B—C7—H7C	109.5	N4—C23—H23B	109.5
C6—C8—H8A	109.5	H23A—C23—H23B	109.5
C6—C8—H8B	109.5	N4—C23—H23C	109.5
H8A—C8—H8B	109.5	H23A—C23—H23C	109.5
C6—C8—H8C	109.5	H23B—C23—H23C	109.5
H8A—C8—H8C	109.5	N4—C24—H24A	109.5
H8B—C8—H8C	109.5	N4—C24—H24B	109.5
Si1—C9—H9A	109.5	H24A—C24—H24B	109.5
Si1—C9—H9B	109.5	N4—C24—H24C	109.5
H9A—C9—H9B	109.5	H24A—C24—H24C	109.5
Si1—C9—H9C	109.5	H24B—C24—H24C	109.5
